# Water as a Source of Antimicrobial Resistance and Healthcare-Associated Infections

**DOI:** 10.3390/pathogens9080667

**Published:** 2020-08-18

**Authors:** Claire Hayward, Kirstin E. Ross, Melissa H. Brown, Harriet Whiley

**Affiliations:** 1 Environmental Health, College of Science and Engineering, Flinders University, Adelaide, South Australia 5042, Australia; kirstin.ross@flinders.edu.au (K.E.R.); harriet.whiley@flinders.edu.au (H.W.); 2 College of Science and Engineering, Flinders University, Adelaide, South Australia 5042, Australia; melissa.brown@flinders.edu.au

**Keywords:** antibiotic resistance, antimicrobial resistance, water, waterborne outbreak, healthcare associated infection, biofilm

## Abstract

Healthcare-associated infections (HAIs) are one of the most common patient complications, affecting 7% of patients in developed countries each year. The rise of antimicrobial resistant (AMR) bacteria has been identified as one of the biggest global health challenges, resulting in an estimated 23,000 deaths in the US annually. Environmental reservoirs for AMR bacteria such as bed rails, light switches and doorknobs have been identified in the past and addressed with infection prevention guidelines. However, water and water-related devices are often overlooked as potential sources of HAI outbreaks. This systematic review examines the role of water and water-related devices in the transmission of AMR bacteria responsible for HAIs, discussing common waterborne devices, pathogens, and surveillance strategies. AMR strains of previously described waterborne pathogens including *Pseudomonas aeruginosa, Mycobacterium* spp., and *Legionella* spp. were commonly isolated. However, methicillin-resistant *Staphylococcus aureus* and carbapenem-resistant Enterobacteriaceae that are not typically associated with water were also isolated. Biofilms were identified as a hot spot for the dissemination of genes responsible for survival functions. A limitation identified was a lack of consistency between environmental screening scope, isolation methodology, and antimicrobial resistance characterization. Broad universal environmental surveillance guidelines must be developed and adopted to monitor AMR pathogens, allowing prediction of future threats before waterborne infection outbreaks occur.

## 1. Introduction

Healthcare-associated infections (HAIs) are defined as infections caused as a direct or indirect result of an individual receiving healthcare [1]. This may occur in hospitals, aged care facilities, dental clinics and long-term care facilities [2]. The United States (US) Centers for Disease Control and Prevention (CDC) have estimated that 1 in 25 hospital patients are diagnosed with a HAI each year [3]. Additionally, there are over 4 million HAIs in Europe, 1.7 million in the US and 165,000 in Australia annually [4]. HAIs result in unnecessary morbidity and mortality with estimates from the US indicating HAIs are responsible for approximately 99,000 unnecessary deaths every year [4]. Hospital patients and aged care residents are especially vulnerable to infection due to their potentially compromised immune systems [5]. HAIs are commonly associated with catheters, surgical sites and ventilators [6], where the causative organisms may originate from the patient’s own microbial flora, other patients, staff or from the healthcare facilities physical environment [5]. The US CDC have identified a number of causative agents that pose serious threats to hospitalized patients including *Acinetobacter* spp., influenza, *Klebsiella* spp., methicillin-resistant *Staphylococcus aureus* (MRSA), *Clostridium difficile, Pseudomonas aeruginosa,* non-tuberculous mycobacteria (NTM) and norovirus [7]. The significance and severity of HAIs are increasing due to the rise in antimicrobial resistance and emergence of multidrug resistance (MDR) [8]. Thus, treatment for patients suffering HAIs resistant to traditional antibiotic therapies is more precarious, costly and, in the worst case scenario, unsuccessful [6]. The increase in antimicrobial resistance is driven, in part, by the inappropriate use of antibiotics and ineffective disinfectant protocols [9]. Understanding potential environmental reservoirs of infectious bacterial species is needed to develop and implement effective infection control [1]. Strategies for the prevention of person-to-person transmission are well defined, including disinfection procedures of dry surface fomites such as bed rails, doorknobs and light switches [1,10,11,12]. However, there are limited studies investigating the role of environmental microorganisms, including waterborne pathogens such as *Legionella* spp., *P. aeruginosa* and *Mycobacterium* spp. [13,14,15,16,17,18,19,20,21,22,23,24,25,26,27]. It has been estimated that 20% of nosocomial pneumonias are caused by waterborne *P. aeruginosa* in the US, resulting in a conservative annual mortality of approximately 1400 individuals [28]. An outbreak of *L. pneumophila* infection in the neonatal unit of a private hospital was linked to a cold-mist humidifier filled with contaminated tap water, resulting in nine infections and three deaths [29]. Transmission of these waterborne pathogens may occur via water related devices such as showers, drinking fountains, bathtubs, dental units, ice machine, humidifiers, sinks and toilets [27]. Notably, approximately 80% of chronic and recurrent microorganism infections are caused by biofilms [30], which are communities of microorganisms, providing protection from adverse environmental conditions and antimicrobial agents [30]. 

This systematic review examined the role of water in the transmission of AMR pathogens that are responsible for HAIs. Common waterborne devices, pathogens, and surveillance strategies are discussed. A greater understanding of the ecological niche of these pathogens is needed to develop improved management strategies for the prevention of waterborne HAIs.

## 2. Results

**Figure 1:**
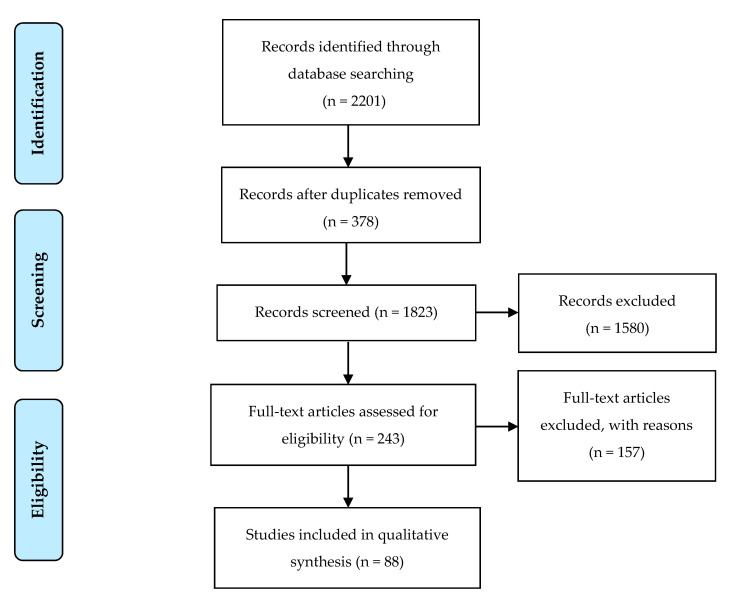
Flow diagram presenting the search strategies used, based on the PRISMA statement reporting guidelines for systematic literature reviews [105].

Two thousand, two hundred, and one papers were retrieved from SCOPUS and Web of Science using the search terms identified (Figure 1). After applying the inclusion and exclusion criteria described in Figure 1, a total of 88 papers were included for review. These were further divided such that 21 papers (presented in Table 1) described studies specifically investigating the presence of AMR bacteria in water and water-related devices including tap faucets, drains, showers, and baths. A further 67 papers that did not specifically investigate water but included some water sampling are presented in the Table S1. These include clinical outbreak investigations and other studies screening a range of environmental sources within healthcare facilities.

### 2.1. Study Sites

Of the 21 papers that specifically investigated the presence of pathogens associated with HAIs in water sources (Table 1), 15 studies were from Europe, 3 from North and South America, 2 from Africa and 1 from Asia. Seventeen studies sampled water sources from hospitals, one from a residential care home, one from dental chair units, one from a medical center and another from a sanatorium. AMR bacterial species were found in potable water samples (15 studies), followed by showers (2 studies) and building water distribution systems (2 studies), sinks (1 study), baths (1 study), haemodialysis water (1 study) and drains (1 study). 

In those clinical outbreak investigations and studies examining a range of environmental sources (Table S1), there were 27 reports from Europe, 22 from Asia, 11 from the Americas, 6 from Africa and 1 published from Oceania. Of these studies, 30/67 found AMR bacterial contamination within a water source, including tap water, hydrotherapy pool water, nasogastric water, and incubator water. Taps and tap components such as aeration grids, tap handles, and hands-free taps had AMR bacterial contamination in 18/67 studies. Sink and sink components such as drain holes, sink surfaces, drainpipe leaks and sink traps were found to have multidrug resistant (MDR) bacterial contamination resistant to two or more antimicrobials in 45/67 studies (Table S1). Shower components such as the shower hoses, showerhead and outlets were contaminated with AMR bacteria in 11/67 studies. Baths were found to have MDR bacterial contamination in 4 studies and bath toys were identified as a source of contamination in 1 study [14,16,52,53,54]. 

### 2.2. Identified Pathogens Associated with HAIs

Seven of the studies used culture-based techniques to investigate the bacterial diversity in the water sources in healthcare facilities. The pathogens identified are detailed in Table 1 and include *Achromobacter* spp., *Acinetobacter* spp., *Acinetobacter anitratus, Acinetobacter baumannii, Acinetobacter haemolyticus, Acinetobacter lwoffi., Aeromonas* spp., *Alcaligenes xylosoxidans, Burkholderia cepacia, Chryseobacterium meningosepticum, Citrobacter* spp., *Citrobacter diversus, Citrobacter freundii, Enterobacter* spp., *Enterobacter aerogenes, Enterobacter agglomerans, Enterobacter cloacae, Erythrobacter* spp., *Escherichia coli, Flavobacterium* spp., *Klebsiella oxytoca, Klebsiella pneumoniae, Leclercia adocarboxylata, Moraxella* spp., *Moraxella osloensis, Mycobacterium* spp., *Novosphingobium* spp., *Pantoea agglomerans, Proteus mirabilis, Pseudomonas* spp., *Pseudomonas acidovorans, P. aeruginosa, Pseudomonas cepacia, Pseudomonas fluorescens, Pseudomonas putida, Pseudomonas stutzeri, Ralstonia picketti, Serratia* spp., *Serratia liquefaciens, Serratia marcescens, Serratia plymuthica, Sphingomonas* spp., *S. aureus,* and *Stenotrophomonas maltophila.*

Fourteen studies investigated the presence of one specific bacterial species or genus that may cause HAIs in water and water-related devices. Of these, seven papers investigated *P. aeruginosa* exclusively, three investigated *Legionella* spp. and two papers focused specifically on *L. pneumophila*. One paper focused on *Enterococci* spp. and another focused on *Sphingomonadacae* spp. (Table 1).

Twenty studies undertook comprehensive environmental bacterial screens of the study sites. These studies included additional pathogens such as *Acidovorax* spp., *Acinetobacter johnsonii, Aeromonas caviae, Aeromonas hydrophila, Alkaligenes faecalis, Bosea* spp., *Chryseobacterium* spp., *Chryseobacterium indologenes, Elizabethkingia meningoseptica, Enterobacter asburiae, Enterococci* spp., *Klebsiella ozenae, Methylobacterium* spp., *Mycobacterium chelonae, Pantoea calida, Proteus* spp., *Proteus vulgaris, Providencia stuartii, Raoultella ornithinolytica, Raoultella planticola, Sphingomonas paucimobilis, Staphylococcus citrus, Staphylococcus epidermidis* and *Staphylococcus* spp., as shown in Table S1. However, due to the design of some studies, it was not always clear whether these bacterial species were isolated from the water samples taken or from other environmental sources.

Thirty-five of 67 (Table S1) investigated bacterial clinical outbreaks in one or more healthcare facilities identified contamination of water and/or a water related device as the likely source of transmission via strain comparison. This included HAI outbreaks of *Achromobacter bacteraemia, Achromobacter denitrificans, Achromobacter xylosoxidans, Acinetobacter bereziniae, A. hydrophila,* carbapenem-resistant *Enterobacteriaceae* (CRE)*,* carbapenem-resistant *E. coli, Citrobacter amalonaticus, C. freundii, Collinsella aerofaciens, Comamonas testosterone, E. cloacae* complex, *Klebsiella* spp., *Pseudomonas medocina, Pseudomonas nitroreducens, Pseudomonas oleovorans* and *P. putida*. A surveillance review of waterborne diseases in the US from 2013 to 2014 found that there were 42 outbreaks from drinking water, resulting in 13 deaths all caused by *Legionella* spp. [55].

Nine studies compared clinical bacterial isolates and environmental isolates, including those from water samples for molecular epidemiology in non-outbreak settings. These studies included bacterial species such as *Aeromonas spp., Burkholderia spp., Klebsiella quasipneumoniae, P. aeruginosa* and *S. maltophila*, as shown in Table S1.

### 2.3. Antimicrobial Resistance of Identified Strains

Several AMR pathogens of concern, as classified by the US CDC, were identified by studies included in this review (Table 1 and Table S1). Specifically, three studies detected CRE, one from a plumbing fixture, one from a water sample and one sample site was unspecified [21,52,56]. MDR *P. aeruginosa* strains were also found in 12 studies, most commonly from potable water samples (7 studies), sinks (3 studies) and faucets (2 studies) [14,15,16,33,39,40,43,49,50,57,58,59]. Eight studies reported AMR *Acinetobacter* spp. of which five reported MDR isolates and one study identified the resistance genes *tetG, ermX* and *ermF* in bacteria within a biofilm sample [32,37,42]. Additionally, the resistance gene OXA-23 was found in *A. baumannii* sampled from hospital water which has been linked to β-lactam antibiotic resistance [60]. Specific genetic elements such as Opr protein-mediated resistance to fluoroquinolone antibiotics was also found in *P. aeruginosa* isolates [37]. MRSA was detected in every bathroom sink tap that was tested in a UK hospital. However, it is unclear which antibiotics this specific environmental isolate was resistant to [61]. Sixteen studies that investigated water and water-related devices found bacterial isolates that were resistant to two or more of the antibiotics that were tested (Table 1). One study investigating *P. aeruginosa, P. stutzeri, B. cepacian* and *A. haemolyticus* in hospital water samples found that all isolates were resistant to seven or more of the 11 antibiotics that were tested, including amikacin, ceftazidime, chloramphenicol, ciprofloxacin, cefepime, gentamicin, imipenem, tetracycline, trimethoprim, tobramycin and piperacillin-tazobactam [33]. One study into the presence of *L. pneumophila* in a hospital hot water system found that the minimum inhibitory concentration (MIC) values were higher in serogroup 1 isolates compared to non-serogroup 1 isolates for the antibiotics azithromycin, ciprofloxacin, levofloxacin, moxalactam and tigecycline [34]. Resistance to β-lactamase inhibitors such as tazobactam and clavulanic acid was identified in *K. oxytoca, P. calida, R. ornithinolytica* and *P. aeruginosa* isolated from hospital sinks, drains, shower heads, water and aerators [15,25,62]. Biofilm samples taken from hospital shower heads contained *Erythrobacter* spp., *Mycobacterium* spp., *Novosphingobium* spp. and *Sphingomonas* spp. isolates that carried the resistance genes *aac2Ib, aac2Ic, aph3Ic, bacA, bL2b, ceoB* and *mfpA* that have been linked to biofilm formation, virulence, peroxide resistance, DNA repair, antibiotic resistance, and antigenic variation traits [45].

### 2.4. Detection Methods

There was significant variation in the methods used for detecting bacterial species from the environment. Fifteen studies (Table 1) examined water using culture techniques. Specifically, eleven studies performed membrane filtration followed by plating onto selective agar media, nine of these studies used 0.45 µm pore diameter filters and two did not specify (Table 1). Of those that specifically investigated *Legionella* spp., two studies referenced the International Organization for Standardization (ISO) 11731—water quality enumeration of *Legionella* [31,36]. One study investigating *L. pneumophila* followed Italian guidelines for prevention and the control of legionellosis [34] and two studies used other culturing techniques [35,44]. Of the studies investigating *P. aeruginosa,* four papers used membrane filtration methods followed by plating onto selective media such as R2A, cetrimide, and Columbia with horse blood, one of which referenced the ISO 16266:2008—detection and enumeration of *P. aeruginosa* specifically [38,39,43,48]. One paper alternatively inoculated malachite-green broth with the individual environmental water sample and subcultured onto cetrimide agar to isolate *P. aeruginosa* [40]. Bacterial species from biofilm and swab samples taken from water-related devices were isolated using a variety of methods including direct inoculation onto cetrimide, MacConkey, tegritol-7, or deoxycholate agar, and centrifugation to resuspend a pellet for inoculation onto selective agar, as shown in Table 1 [33,37,42,45,49]. Five studies used additional methods such as polymerase chain reaction (PCR), matrix-assisted laser desorption/ionization-time of flight (MALDI-TOF), VITEK-2, multiplex PCR and 16S gene sequencing to identify isolated bacterial species (Table S1) [63,64,65].

### 2.5. Antimicrobial Resistance Characterization Methods

A range of methods were used to determine the antimicrobial resistance characteristics of isolated strains. Seventy-one of 88 studies (Table 1 and Table S1) used traditional microbiological methods including disc diffusion (56 studies), agar dilution (4 studies), broth microdilution (5 studies) and E-test strips (6 studies). Other approaches for characterizing antimicrobial resistance included PCR (17 studies) and comparison to known AMR strains using VITEK-2 system (5 studies), pulse field gel electrophoresis (PFGE) (3 studies), microscan (2 studies), microarray (1 study) and multilocus sequencing typing (MLST) (1 study).

Comparing the antimicrobial resistance is challenging due to the varying approaches used in the different studies. A joint initiative by the European CDC and US CDC provided definitions for the terms MDR and XDR to standardize international terminology. To facilitate these definitions, lists of antimicrobial categories and breakpoints were developed from the Clinical Laboratory Standards Institute (CLSI), the European Committee on Antimicrobial Susceptibility Testing (EUCAST) and the United States Food and Drug Administration (FDA). MDR was defined as acquired non-susceptibility to at least one agent in three or more antimicrobial categories. XDR was defined as non-susceptibility to at least one agent in all but two antimicrobial categories [66]. The terms MDR and extensively drug resistant (XDR) were used by five studies and the terminology has been reported as stated in the papers (Table 1 and Table S1); however, it was unclear what specific antibiotics the isolates were resistant to [32,39,49,50,58]. Of the studies detailed in Table S1, 20/67 studies reported the environmental isolates as a whole data set rather than describing the phenotypes of each individual strain.

## 3. Discussion

### 3.1. Water as a Source of HAIs 

Water sources and water-related devices are often contaminated with pathogens responsible for HAIs. This may occur when microorganisms survive treatment protocols or via end point contamination [67]. The design of a hospital or healthcare facility’s water system can influence the risk of microbial contamination [68]. Complex infrastructure may have points of heat transfer and stagnation which can promote biofilm formation, microbial growth and the rise or transfer of antimicrobial resistance [69]. The CDC Antibiotic Resistance Threats Report estimated that there are more than 2.8 million AMR infections each year in the US resulting in approximately 35,000 deaths [8]. This review identified that water and water-related devices play a significant role in the transmission of AMR HAIs with subsequently an economic and health imperative to improve the control of hospital and healthcare water sources.

This review identified a range of waterborne pathogens present in the potable water supply and plumbing surfaces (such as drains and tap faucets). However, pathogens not typically considered waterborne were also detected, including *S. aureus, Moraxella* spp. and *E. aerogenes* [32,42,51,54,70,71,72,73]. For example, AMR pathogens of concern, extended-spectrum beta-lactamase-producing Enterobacteriaceae and MRSA, were located in a hospital sink bowl, hospital bathroom sink taps and a hospital bathtub [51,61,74]. This raises the hypothesis that end point contamination may be occurring from patient-to-water source. A study examining the influence of contaminated splash backs when handwashing in twenty faucet/sinks in hospital intensive care units found that the faucet spouts were more contaminated than the sink bowl and drains. Flawed sink design such as shallow bowls enable splashing contaminated sink contents onto patient care items, healthcare workers hands and the patients’ broader environment [75].

Numerous approaches are taken to ensure a facility’s potable water supply is suitable for human use and consumption. The Healthcare Infection Control Practices Advisory Committee (HICPAC) has published guidelines to prevent the growth of bacterial species such as *Legionella* spp. [69]. This includes recommendations such as maintaining adequate water pressure, temperature and preventing stagnation. Some older healthcare facilities, built prior to such guidelines, often have plumbing infrastructure that doesn’t meet these requirements. If infrastructure recommendations can’t be met, additional measures such as chlorine treatment, copper-silver ionization or ultraviolet light can be used to ensure water quality [76]. As municipal water passes through the distribution network, the amount of residual disinfection agent can vary. If the facility is far away from the point of disinfection, the water the building receives may have disinfectant levels lower than the effective concentration [69]. The success of disinfection approaches may also be impacted by resistant species. For example, copper resistant *P. aeruginosa* was isolated from a French water system and tap aeration grids, and hydrogen peroxide and silver nitrate resistant *Legionella* spp. were isolated from a hospital’s water supply [57,77]. Future work is needed to inform and improve HAI guidelines regarding the use of water and prevent the spread of AMR pathogens. 

### 3.2. Biofilm Formation and Antimicrobial Resistance

Biofilms are secure, often heterogeneous, communities of microorganisms which colonize and grow on surfaces of medical implants, plumbing infrastructure and on patients [30]. They are comprised of dense microbial populations immobilized by an extracellular matrix comprised of bacterial secreted polymers such as exopolysaccharides (EPS), extracellular DNA and proteins [30]. Recently, point of use filters have been implemented in healthcare facilities as an additional form of protection from bacteria present in the water supply [78]. Even though *P. aeruginosa* and *Legionella* spp. were eliminated from taps in an intensive care unit in Hungary when point of use filters were installed, decreasing cases of infection to zero [79], they have been found to facilitate biofilm formation inside the filter when not maintained correctly, directly affecting the bacterial load in the water over time [78,80]. Within hospital water distribution systems and plumbing fixtures, biofilms provide a source of nutrients and protection from disinfection processes [30]. Biofilm growth is promoted in areas of low flow rate and stagnation which allows for bacterial attachment to the infrastructure surface [81].

The metabolic activity of the bacterial biofilm communities is different compared to planktonic bacteria, such as increased rates of EPS production, activation or inhibition of genes associated with biofilm formation and decreased growth rate [30]. The role of EPS has been linked to conferring tolerance to aminoglycosides by quenching their activity via a diffusion reaction inhibition [82]. An outbreak strain of aminoglycoside resistant *P. aeruginosa* was found on a contaminated bath toy in an Australian hospital [16]. Biofilm production confers protection to the microorganism communities from harmful pH, osmolarity, nutrient scarcity and shear forces [30]. Bacteria in biofilms are also more resistant to antimicrobial exposure by blocking the access of antibiotics, increasing the resistance by up to 1000-fold when compared to planktonic bacteria [45]. Once a biofilm community has reached maturation, species such as *L. pneumophila* may enter a viable non-culturable (VBNC) stationary phase as a way of surviving antibiotic stress [30,83]. Recent data suggests that hot water flushing and chlorination are not effective in eliminating *Legionella* spp. from plumbing systems over long periods of time [76,84]. This may be due to in part to bacterial species such as *Legionella* spp. being intracellular parasites of free living amoeba, resulting in conferred protection from disinfection by techniques when phagocytized [76].

One of the predominant mechanisms for acquiring antimicrobial resistance is uptake of resistance genes by horizontal gene transfer (HGT) [82]. The high cell density and presence of genetic elements from a highly heterogeneous community promotes this transfer via mechanisms such as conjugation, transformation or transduction [82]. Antimicrobial resistance may also be acquired via a mutation event in a bacterial chromosome [85]. Once the resistance mutation has stabilized in a generation, it will be directly transmitted to all descendant cells by mitosis [86]. This process is known as vertical transmission. Under antimicrobial stress, resistance may arise via a combination of both HGT and vertical transmission. These genetic elements may enhance antimicrobial defense strategies by restricting drug entry via modifications to the cell wall, pumping the drug out of the cell, enzymatic degradation of the drug or deleting or decreasing the affinity of the involved target [87]. Exposure to chlorine can also stimulate the expression of efflux pumps and drug resistance operons, as well as induce mutations in some genes leading to increased antimicrobial resistance [45]. Some antibiotic gene profiles observed in hospital shower hose metagenomes have been reported to be triggered by biocide exposure [45,88,89]. These include commonly used antibiotics such as chloramphenicol, kanamycin, and penicillin. Species such as *Mycobacterium* spp. are commonly found in biofilm communities [45]. This may be because of physiochemical properties such as plumbing pipes being galvanized or made of copper, the disinfectant use and low organic carbon content of the water selectively favoring the growth of some *Mycobacterium* spp. [45]. When exposed to stress conditions, *Mycobacterium* spp. can modify the cell membrane fatty acid composition producing an altered permeability to biocide and antibiotic compounds [45,90,91]. The biofilm-forming capacity of pathogens such as *P. aeruginosa, Mycobacterium* spp. and *S. maltophilia* can promote the attachment of other pathogens such as *Salmonella* spp., *Campylobacter* spp. and *S. aureus* that are typically found in the wider hospital environment [92].

### 3.3. Detection Methods

#### 3.3.1. Outbreak Investigations

Environmental screening typically takes place in response to an outbreak rather than as routine sampling, which leads to inconsistencies between the types of samples taken, isolation methods and antimicrobial resistance reporting. Thirty-five of 88 papers included in this review explored clinical outbreaks and sampled water and/or water related devices as a part of the investigation (Table S1). In contrast, 20/88 papers conducted broad screens of the facilities’ environment in a non-outbreak setting. The Australian Guidelines for the Prevention and Control of Infection in Healthcare suggest that environmental testing should be carried out to identify risk factors [1]. However, it is not clear what sampling techniques are to be used and which samples should be taken [1]. Similarly, in the UK, there is guidance available from The National Specifications for Cleanliness in the NHS for monitoring the hospital environment. However, there was no indication of microbiological screening [93,94]. The absence of a standard approach for when environmental sampling should occur and what samples should be taken limits data comparisons that can be made and potentially overlooks reservoirs such as water and water-related devices.

#### 3.3.2. Pathogen Detection from Environmental Sources

International standards have been published for the processing of environmental water samples for organisms such as *Legionella* spp., *P. aeruginosa* and *E. coli*. However, of the publications reviewed in this study, only three referenced a specific ISO standard [31,36,43]. There was significant variation between sampling techniques and selective growth media used in publications that investigated water-related surfaces such as tap faucets and drain holes [32,33,39,40,41,43,47,48,50,51]. Traditional microbial culturing techniques used for waterborne pathogens such as *Legionella* spp. has presented challenges for some environmental samples as VBNC cells and result in false negative results [83,95]. Furthermore, environmental waterborne pathogens often adapt to environments that are nutrient poor, which may be difficult to culture on nutrient-rich media types. Using nutritionally reduced media types such as R2A agar for longer incubation periods (14–28 days) may enhance the recovery of chlorine damaged and stressed bacteria [76]. Environmental water samples are often passed through membrane filters to concentrate and isolate any bacterial cells present in the sample. The pore diameter in these membrane filters typically ranges from 01 to 0.45 µm depending on the intended use [96]. The size, shape and biovolume of bacteria may influence the filterability of a sample and potentially lead to inaccurate findings, particularly if multiple species of bacteria are being investigated using the one pore diameter [96]. Alternative molecular techniques for bacterial detection such as qPCR and whole-genome sequencing (WGS) have been employed by 27 studies included in this review [13,14,17,18,20,25,26,34,37,40,43,45,46,51,52,56,57,59,60,63,64,65,97,98,99,100,101]. Molecular techniques have significant advantages such as rapid turnaround times and detection of non-culturable cells [102]. However, limitations such as environmental inhibitors and potential overestimation of bacterial presence due to the amplification of non-viable cells needs to be considered [103]. For some bacteria, PCR-based techniques have been developed to differentiate viable cells from dead cells. For example, ethidium monoazide bromide viability staining can be used in conjunction with qPCR to enumerate viable cells (such as *L. pneumophila)* [102]. In order to implement effective surveillance programs, detailed and consistent sampling techniques and detection methods are essential.

#### 3.3.3. Characterizing AMR

International standards for antimicrobial susceptibility testing have been jointly published by the Clinical and Laboratory Standards Institute and the European Centre for Disease Prevention and Control and the US CDC [104]. These standards include antibiotics to be tested against species that have commonly been associated with HAIs including *Acinetobacter* spp., *P. aeruginosa,* and *S. aureus* as well as breakpoints to determine an isolate’s resistance to each antibiotic. Irrespectively, the reporting of resistant species remains inconsistent. When papers report the resistance profiles of an AMR isolate using differing units such as µg/mL or mg/mL MICs, percentage of isolates resistant or as specific resistance genes, the comparisons that can be made between studies are limited to broad comments rather than quantifiable data trends.

## 4. Materials and Methods

This systematic literature review is based on an adapted version of the PRISMA statement [105], presented in Figure 1. This tool is an evidence-based system for evaluating and reporting evidence. A systematic search of the SCOPUS and Web of Science databases was performed, and all literature published prior to 2020 was included. Keywords used in this search are presented in Table 2. A detailed search strategy was established to ensure a comprehensive literature review of all identified antimicrobial resistance bacteria in healthcare water environments was achieved.

All titles and abstracts of published literature were manually reviewed to ensure that they reported antimicrobial resistant bacteria to the genus level. The paper must also have reported this presence in a healthcare setting water source or water-related device. Papers were excluded if they were not written in English, reviews, reports of human clinical infection with no mention of a contributing water source, laboratory setting experiments and wastewater investigations. All relevant papers had key points taken and recorded including the study site, water source, country, species of organism, isolation method used, antimicrobial method used, and relevant characteristics.

## 5. Conclusions

Although environmental reservoirs such as dry surface fomites have been identified as potential sources of HAIs, water and water-related devices are often overlooked. Understanding the role that water and water-related devices play as reservoirs for AMR bacteria is imperative to prevent transmission pathways that may cause HAIs. Water sources contaminated with AMR pathogens provide unique environments for the dissemination of antimicrobial resistance genes that are often unaffected by commonly employed disinfection strategies. Sinks, tap faucets, drains, bathtubs, drinking water fountains, aeration grids, showers and haemodialysis water have all been identified as contaminated with one or more species of AMR bacteria capable of causing HAIs. Broad universal environmental surveillance guidelines must be developed, including sampling locations, methodology and resistance reporting, to monitor resistant pathogens and predict future threats before infection outbreaks occur. By understanding how water and water related devices may harbor AMR species, better environmental controls can be implemented to significantly reduce the rates of waterborne HAIs.

## Figures and Tables

**Table 1:** Summary of reports and studies identifying antimicrobial resistant bacterial species within healthcare water sources and water-related devices.

Study Site	Reservoir	Organism	Country *	Bacterial Isolation Methods ^◊^	Antimicrobial Methods ^†^	Antimicrobial Characteristics	Additional Comments ^×^	Reference
Hospital	Water	*Legionella* spp.	Greece *	ISO 11731 (filtration, untreated, heat and acid treatments) plated on GVPC agar	E-test strips	Five strains displayed low-level resistance to CIP and ERY	SGs 1–15 identified. Antibiotics tested: CIP, ERY	[31]
Hospital	Water	*Burkholderia cepacia Pseudomonas stutzeri* *Chryseobacterium meningosepticum Stenotrophomonas maltophilia* *Enterobacter cloacae Acinetobacter baumannii* *Escherichia coli* *Proteus mirabilis* *Alcaligenes xylosoxidans* *Pseudomonas aeruginosa* *Pseudomonas putida* *Serratia liquefaciens Moraxella osloensis* *Serratia plymuthica*	Greece	Membrane filtration and plated on m-endo medium and cetrimide agar	Agar dilution	*S. maltophila* isolate resistance: 37% resistant to CAZ 58% resistant to FEP 100% resistant to IPM *E. coli* isolates: 55% resistant to TIC *P. mirabilis, P. putida, S. liquefaciens, P. stutzeri* and *S. plymuthica* exhibited resistance to tetracycline 19% of the total enterobacteria and 35% of the total non-fermenting isolates were MDR	Antibiotics tested: AMK, CAZ, CIP, FEP, IPM, TET, TIC, SXT, TOB	[32]
Hospital	Water	*Acinetobacter haemolyticus* *B. cepacia* *Pseudomonas aeruginosa* *P. stutzeri*	Brazil	MPN, APHA 2000 plated on MacConkey agar	Disc diffusion	*B. cepacian* isolates showed resistance to 10/11 antibiotics *P. aeruginosa* isolates showed resistance to 11/11 antibiotics *A. haemolyticus* isolates showed resistance to 11/11 antibiotics *P. stutzeri* isolates showed resistance to 7/11 antibiotics	Antibiotics tested: AMK, CAZ, CCHL, CIP, FEP, GEN, IPM, TET, TMP, TOB, TZP	[33]
Hospital	Hot water system	*Legionella pneumophila*	Italy	Italian guidelines for prevention and control of Legionellosis	VITEK-2	MIC values of *L. pneumophila* SG 1 were higher than non-SG 1 isolates for AZI, CIP, LEV, MOX, and TIG No difference in MIC values between SGs for CEF, CLA, DOX, ERY, and RIF	Antibiotics tested: AZM, CIP, CLR, CTX, DOX, ERY, LVX, MXF, RIF, TGC	[34]
Hospital	Water system	*Legionella* spp.	Turkey	Culture methods	Broth dilution	MICs: Greatest MIC to CLR	Antibiotics tested: AZM, CIP, CLR, LVX, RIF	[35]
Hospital	Water	*L. pneumophila*	Spain	UNE-ISO 11731:2007 (filtration: untreated, acid and heat treatments) plated on GVPC agar	E-test strips, Disc diffusion	E-test strips: Greatest average MIC resistance from CIP and DOX Lowest average MIC resistance from AMC and AZT Disc diffusion: Greatest average disc inhibition from AZT and AMC Lowest average disc inhibition from SXT and RIF	Antibiotics tested: E-test strips: AMC, AZM, CIP, CTX, DOX, ERY, LVX, MXF Disc diffusion: AMC, AZM, CIP, CTX, ERY, FOX, LVX, MXF, RIF, SXT	[36]
Hospital	Water	*Acinetobacter* spp. *Aeromonas* spp. *Citrobacter* spp. *Enterobacter* spp. *Escherichia coli* *Klebsiella oxytoca* *Klebsiella pneumoniaeLeclercia adocarboxylata* *Pseudomonas* spp. *Serratia* spp.	Turkey	Membrane filtration and inoculated in MacConkey broth and MacConkey agar	Disc diffusion PCR	*E. coli* isolates: 1 isolate resistant to CRO 5 isolates resistant to AMP 1 isolate resistant to PIP Other species: 3 *Pseudomonas* spp. isolates showed resistance to CAZ, IMP and GEN	Antibiotics tested: AMC, AMK, AMP, CAZ, CEF, CHL, CIP, CRO, FEP, FOX, GEN, IPM, MEM, PIP, TET, SXT, TZP	[37]
Hospital	Water	*P. aeruginosa*	France	Membrane filtration	Disc diffusion	Copper tolerant isolates.	Antibiotics tested: AMK, ATM, CAZ, CIP, FEP, FOF, IPM, MEM, TOB, TZP	[38]
Hospital	Water	*P. aeruginosa*	India	Membrane filtration. Plated on R2A agar immediately and on either cetrimide, Columbia + 5% horse blood or R2A after 14 days	Disc diffusion	All isolates showed resistance to TET and PEN 2 isolates resistant to STR 4 isolates resistant to NET 5 isolates showed MDR	Antibiotics tested: NET, OFX, PEN, STR, TET	[39]
Hospital	Water	*P. aeruginosa*	Tanzania	Water sample inoculated directly in malachite-green broth then subcultured on blood and cetrimide agar	VITEK-2	Resistance (% of isolates): ETP (2.6%); IPM (2.6%); TZP (2.6%); TOB (5.1%); GEN (12.8%); CIP (15.4%); PIP (18%); FOF (61.5%); ATM (100%)	Antibiotics tested: AMK, ATM, CAZ, CIP, CST, ETP, FEP, FOF, GEN, IPM, MEM, PIP, TOB, TZP Two hospitals sampled; one received water from a deep drilled well and the other from Lake Victoria	[40]
Hospital Dental chair	Water	*Sphingomonadacae* spp.	Portugal *	Membrane filtration and plated on R2A, GSP, *Pseudomonas* isolation and tergitol-7 agar	ATB PSE EU system	Hospital taps resistance (% of isolates): TIM (2%); CIP (11%); MEM (17%); CAZ (21%); FEP (26%); TSU (30%); TIC (36%); TOB (36%); LVX (42%); FOS (42%); PIP (49%); TZP (36%); CST (94%) Dental chair resistance (% of isolates): TZP (17%); CAZ (17%); MEM (17%); TOB (17%); TSU (17%); FEP (33%); GEN (33%); CIP (33%); TIC (50%); PIC (67%); COL (83%)	Antibiotics tested: CAZ, CIP, CST, FEP, FOF, GEN, IPM, LVX, MEM, PIP, TIC, TIM, TOB, TSU, TZP	[41]
Medical centre	Drain	*Achromobacter* spp. *Acinetobacter anitratus* *Acinetobacter lwoffi* *Aeromonas* spp. *Enterobacter agglomerans* *Enterobacter cloacae* *Flavobacterium* spp. *Moraxella* spp. *Pseudomonas acidovorans* *P. aeruginosa* *Pseudomonas* spp. *Pseudomonas cepacia* *Pseudomonas fluorescens* *Pseudomonas putida* *P. stutzeri* *Stenotrophomonas maltophila*	USA	Drains swabbed and plated on deoxycholate agar biplate with GEN and AMK	Selective media	Resistance (% of isolates): AMK (77%); GEN (88%)	Antibiotics tested: AMK, GEN	[42]
Hospital Residential care home	Taps Shower Drinking fountain	*P. aeruginosa*	Italy	UNI EN ISO 16266:2008. Membrane filtration and plated on Pseudomonas agar with CN supplement	Disc diffusion PCR DNA sequencing	7.72% resistant to imipenem. 13.2% resistant to >1 antibiotic	Antibiotics tested: AMK, ATM, CAZ, CIP, DOR, FEP, GEN, IPM, LVX, MEM, NET, PIP, TIC, TIM, TOB, TZP	[43]
Hospital Sanatorium	Water	*Legionella* spp.	Poland	Culture methods	E-test strips	*L. pneumophila* SG2-14 isolated from one sanatorium showed resistance to AZM	Antibiotics tested: AZM, CIP, RIF	[44]
Hospital	Shower head	*Erythrobacter* spp. *Mycobacterium* spp. *Novosphingobium* spp. *Sphingomonas* spp.	USA	Biofilm removed from inner surfaces and resuspended to be plated on R2A agar	High-throughput sequencing	Resistance genes found: *aac2ib* *aac2ic* *aph3ic* *baca* *bL2b* *ceob* *mfpa*	N/A	[45]
Hospital	Tap water	*P. aeruginosa*	France *	Hospital standard—culture method	Disc diffusion PGFE	7 isolates have Opr-mediated resistance to IPM	Antibiotics tested: CAZ, IPM, PIP Samples taken before and after ICU move for comparison	[46]
Hospital	Haemodialysis water Tap water	*Enterococci* spp.	Greece	Membrane filtration	Agar diffusion	Resistance (% of isolates): RIF (43%) STR (60%) 1 isolate resistant to ERY	Antibiotics tested: AMC, AMP, CIP, ERY, GEN, RIF, STR, TMP, VAN	[47]
Hospital	Water	*P. aeruginosa*	France	Membrane filtration and plated on cetrimide agar	Disc diffusion	*P. aeruginosa* resistant to chlorine disinfection treatment	Antibiotics tested: AMK, CAZ, CTX, FOF, GEN, IPM, OFX, CIP, RIF, TIM, TOB	[48]
Hospital	Sink U-bend	*P. aeruginosa*	France	U-bend content collected and centrifuged pellet was streaked on cetrimide agar	Disc diffusion	Strains: ST1725 (2 MDR isolates) ST539 (100% resistant to IMI) ST1416 (2 MDR isolates) ST540 (1 MDR isolate) STI11 (100% resistant to IPM, 9 MDR isolates) ST622 (7 MDR isolates) ST520 (100% resistant to IPM, 1 MDR isolate)	Antibiotics tested: AMK, CAZ, CIP, FEP, GEN, IPM, MEM, TIC, TOB, TZP	[49]
Hospital	Tap water	*P. aeruginosa* *P. fluorescens* *Ralstonia picketti* *S. maltophila*	Italy	Membrane filtration and placed on cetrimide agar	ATB PSE 5	*P. aeruginosa:* 17 strains non-MDR 4 MDR 3 XDR *S. maltophila:* 1 strain non-MDR 8 strains MDR *P. fluorescens:* 1 MDR strain	Antibiotics tested: AMK, AMP + SUL, CAZ, CIP, CST, FEP, FOF, GEN, IPM, MEM, SXT, TIM, TOB, TZP	[50]
Hospital	Bathtub Tap water	*Citrobacter diversus* *Citrobacter freundiii* *Enterobacter aerogenes* *E. cloacae* *E. coli* *K. pneumoniae* *Pantoea agglomerans* *P. aeruginosa* *Serratia marcescens* *Staphylococcus aureus*	Zambia	Swabs of bathtub and cultured on agar	PCR	MRSA found on bathtubs	Comparison of clinical isolates collected at the same time	[51]

* In countries where the study location was not specified in the article, it was assumed that the country of origin was denoted by the country of the authors. ^◊^ Abbreviations: American Public Health Association, APHA; Glycine Vancomycin Polymyxin Cycloheximide agar, GVPC; International Organization for Standardization, ISO; most probable number, MPN; Spanish Organization for Standardization, UNE ISO. † Abbreviations: BioMerieux susceptibility test, ATB-PSE-EU; polymerase chain reaction, PCR; pulse gel field electrophoresis, PGFE; BioMerieux identification and antibiotic susceptibility testing instrument, VITEK-2. ^×^ Abbreviations: extended-spectrum beta-lactamase, ESBL; multidrug resistant, MDR; minimum inhibitory concentration, MIC; methicillin-resistant *Staphylococcus aureus,* MRSA; serogroup, SG; extensively drug resistant, XDR. Antimicrobial abbreviations: AMK, amikacin; amoxicillin-clavulanic acid, AMC; ampicillin, AMP; azithromycin, AZZM; aztreonam, AZM; aztreonam, ATM; cefepime, FEP; cefotaxime, CTX; cefoxitin, FOX; ceftazidime, CAZ; ceftriaxone, CRO; cephalothin, CEF; chloramphenicol, CHL; ciprofloxacin, CIP; clarithromycin, CLR; colistin, CST; doripenem, DOR; doxycycline, DOX; ertapenem, ETP; erythromycin, ERY; fosfomycin, FOF; fusidic acid, FA; gentamicin, GEN; imipenem, IPM; levofloxacin, LVX; meropenem, MEM; methicillin, MET; moxifloxacin, MXF; neomycin, NEO; netilmicin, NET; ofloxacin, OFX; penicillin, PEN; piperacillin, PIP; piperacillin-tazobactam, TZP; rifampin, RIF; streptomycin, STR; tetracycline, TET; ticarcillin, TIC; ticarcillin-clavulanic acid, TIM; tigecycline, TGC; tobramycin, TOB; trimethoprim, TMP; trimethoprim-sulfamethoxazole, SXT; vancomycin, VAN; sulbactam, SUL; methylisothiazolinone, MIT; tributyl tetradecyl phosphonium chloride, TTPC; didecyldimethylammonium chloride, DDAC; 2,2-dibromo-3-nitrilopropionamide, DBNPA; hydrogen peroxide + silver nitrate, H_2_O_2_ + AgNO_3_; tetrakis (hydroxymethyl)phosphonium sulfate, THPS; sodium hypochlorite, NaOCl; benzalkonium chloride, BZK; cotrimoxazole, TSU; mupirocin, MUP.

**Table 2:** Complete search strategy and all keywords used to identify relevant literature.

Search Terms Employed to Identify Relevant Literature
“antibiotic resistance *”OR “antimicrobial resistance *” OR disinfectant * OR AMR
AND Water OR potable OR drinking OR taps OR faucet OR bath OR shower OR drain OR bathroom OR sink
AND Hospital OR healthcare OR “aged care” OR ICU OR “intensive care unit” OR nosocomial OR HCAI OR “healthcare acquired infection” OR HAI OR “hospital acquired infection” OR “hospital associated infection” OR “healthcare associated infection”

‘*’ Indicates wildcard symbol used to when variations of the search term may be possible.

## Supplementary Materials

The following are available online at https://www.mdpi.com/2076-0817/9/8/667/s1, Table S1: Summary of reports and studies identifying AMR bacterial species within healthcare water sources and water-related devices during outbreak investigation, environmental screening, and molecular epidemiology.
