# Supplementary material for: Water as a Source of Antimicrobial Resistance and Healthcare-Associated Infections
Source: Pathogens. 2020 Aug 18;9(8):667. doi: 10.3390/pathogens9080667 (PMC7459458; doi:10.3390/pathogens9080667)
Supplement: Supplementary file 1 [file pathogens-09-00667-s001.pdf]

Table S1. Summary of reports and studies identifying AMR bacterial species within healthcare water sources and water-related devices during outbreak investigation, environmental screening, and molecular epidemiology.

Study Site	Reservoir	Organism	Country*	Study Type	Antimicrobial Methods ∞	Antimicrobial Characteristics ∧	Reference
Hospital	Water<br>Sink<br>Drain	*P. aeruginosa*<br>*Achromobacter xylosoxidans*<br>*Collinsella aerofaciens*<br>*Pseudomonas putida*<br>*Stenotrophomonas maltophila*<br>*P. medocina*<br>*C. testosterone*<br>*Sphingomonas spp.*	France	Outbreak investigation	Agar dilution, PCR	blaIMP-19 found in *P. aeruginosa, A. xylosoxidans, A. aegrifaciens, P. putida, S. maltophila, P. mendocina, C. testosteroni and Sphingomonas spp.*	[13]
Hospital	Sink<br>Drain<br>Bath<br>Shower	*P. aeruginosa*	USA*	Outbreak investigation	Broth microdilution, PGFE	Bathtub isolate resistant to GEN, TOB, TIC and PIP	[14]
Hospital	Water	*P. aeruginosa*	France	Outbreak investigation	Agar diffusion	Resistant to: TIC, TZP, PIP, CAZ, CIN, FEP, CPR, ATM, GEN, TOB, NET, CIP	[15]
Hospital	Water<br>Sink<br>Faucets<br>Shower hoses<br>Bath toys	*P. aeruginosa*	Australia	Outbreak investigation	Agar dilution, E-test	Aminoglycoside-resistant *P. aeruginosa* isolated from bath toys	[16]
Hospital	Sink<br>Water	*P. aeruginosa*	Mexico*	Outbreak investigation	Vitek-2, PFGE	Resistance profile for environmental isolates not reported individually	[17]
Hospital	Sink	*P. aeruginosa*	Columbia	Outbreak investigation	Microscan, PCR	Resistance profile: IMI and positive for blaVIM	[18]
Hospital	Sink<br>Water	*P. aeruginosa*	India*	Outbreak investigation	Disc diffusion	Resistance reported as whole environmental data	[19]
Hospital	Shower<br>Sink<br>Tap	*P. aeruginosa*	Italy	Outbreak investigation	Vitek-2	Resistance profile for environmental samples not reported	[20]
Hospital	Water<br>Faucets<br>Sink<br>Drain	*P. aeruginosa*	Germany	Outbreak investigation	Broth dilution	Carbapenem resistant isolates found	[21]
Hospital	Sink<br>Shower	*P. aeruginosa*	Finland	Outbreak investigation	Disc diffusion	Environmental resistance profiles reported as total with clinical isolates	[22]

Hospital	Water	*M. chelonae*	Mexico	Outbreak investigation	Broth dilution	Resistance reported as whole environmental data	[23]
Hospital	Drain	*P. aeruginosa*	Sweden	Outbreak investigation	Selective culture	12 drain isolates positive for pae-MBL gene	[24]
Hospital	Sinks	*P. aeruginosa*<br>*E. cloacae*<br>*P. putida*<br>*S. maltophilia*<br>*C. freundii*<br>*Achromobacter denitrificans*<br>*Acinetobacter bereziniae*<br>*A. hydrophila*<br>*Citrobacter amalonaticus*<br>*Pseudomonas nitroreducens*<br>*Pseudomonas oleovorans*<br>*S. marcescens*	Germany	Outbreak investigation	Disc diffusion, E-test, PCR	Resistance to AMP, SAM, PIP, TZP, CRO, CAZ, IMI, MEM, GEN and CIP	[25]
Hospital	Sink	*P. aeruginosa*<br>*P. putida*	UK	Outbreak investigation	Disc diffusion, Whole genome sequencing	Two sink isolates contained *gyrA* and *parC* genes	[26]
Hospital	Sink<br>Drain<br>Shower<br>Bath<br>Toilet	*K. pneumoniae*<br>carbapenemase-Producing *E. coli*<br>Carbapenem-resistant *Enterobacteriaceae*	United Kingdom	Outbreak investigation	PCR, Genome sequencing	CRE positive isolates from plumbing sites	[52]
Hospital	Water<br>Aerator<br>Bathtub	*Sphingomonas paucomobilis*	Turkey*	Outbreak investigation	E-test	2 isolates resistant to CIP and GEN	[53]
Hospital	Sink<br>Bath<br>Shower	*S. aureus*	USA	Outbreak investigation	Disc diffusion	Shower isolates positives for mupirocin-resistant *S. aureus*	[54]
Hospital	Incubator water	*K. pneumoniae*	China	Outbreak investigation	Disc diffusion, PCR	One CRE isolate found in water sample	[56]
Hospital	Water system<br>Tap aeration grids	*P. aeruginosa*	France	Hospital environmental isolates v broad environmental isolates	Vitek-2, PCR	All strains have genes *mer-A* like gene, *czcA*, *copA* and *copB*. Isolates demonstrated resistance to Zn, Cu, Cd and Hg	[57]
Hospital	Faucet	*P. aeruginosa*	China*	Clinical isolates v environmental isolates	Disc diffusion	6 MDR resistant strains	[58]
Hospital	Sink	*P. aeruginosa*	Palestine	Clinical isolates v environmental isolates	Disc diffusion, PCR	Resistance to TET, KAN.<br>*exoT*, *exoS* and *exoY* detected by PCR	[59]
Hospital	Water	*A. baumannii*	Iran	Environmental screen	Disc diffusion, PCR	Resistance to CAZ and GEN.<br>OXA-23 found in one water sample	[60]

Hospital	Taps	MRSA	UK	Treatment efficacy investigation	Disc diffusion	MRSA recovered from every bathroom tested	[61]
Hospital	Sink<br>Tap handle<br>Drain<br>Shower head<br>Aerator	*P. aeruginosa*<br>*Enterobacter asburiae*<br>*Aeromonas caviae*<br>*Aeromonas hydrophila*<br>*Raoultella planticola*<br>*Raoultella ornithinolytica*<br>*C. freundii*<br>*Pantoea calida*<br>*K. oxytoca*<br>*E. cloacae*<br>*K. pneumoniae*	United Kingdom	Environmental screen	N/A	*K. oxytoca* resistant to: GEN, CIP, TZP, CTX, CPD and STR.<br>*P. calida* resistant to: GEN, CIP, CTX and STR.<br>*R. ornithinolytica* resistant to: GEN, CIP, TZP, CTX and CPD.	[62]
Hospital	Drain<br>Taps	*K. pneumoniae*	Norway	Outbreak investigation	E-test, PCR	4 blaKPC-positive *K. pneumoniae* isolates	[63]
Hospital	Drain<br>Water	*Klebsiella quasipneumoniae*	US	Clinical isolates v environmental isolates	Whole genome sequencing	Resistance genes found: blaKPC, *fosA*, blaOKP and oqxA-opxB	[64]
Hospital	Water	*Achromobacter bacteraemia*	France	Outbreak investigation	Disc diffusion, PCR	Resistance reported as whole environmental data	[65]
Hospital	Sink	*Staphylococcus aureus*	Kenya	Outbreak investigation	N/A	Resistance profile of sink isolates not reported	[70]
Hospital	Sink	*P. aeruginosa*<br>*K. pneumoniae*<br>*E. coli*<br>*Proteus* spp.<br>*Staphylococcus citrus*<br>*S. aureus*<br>*Citrobacter* spp.<br>*Staphylococcus epidermidis*<br>*Enterobacter* spp.<br>*B. cepacia*<br>*Alkaligenes faecalis*	United Arab Emirates *	Environmental screen	Disc diffusion	Resistance reported as whole environmental data	[71]
Hospital	Tap	*Staphylococcus* spp.<br>*S. aureus*	Italy	Environmental screen	E-test	Resistance reported as whole environmental data	[72]
Hospital	Tap	*Enterobacter* spp.<br>*K. pneumoniae*<br>*Proteus vulgaris*<br>*S. marcescens*<br>*S. aureus*	Uganda	Environmental screen	Disc diffusion	Resistance reported as whole environmental data	[73]
Hospital	Sink	MRSA	USA	Treatment efficacy investigation	Disc diffusion	Resistance profile data not reported	[74]

Hospital	Water	*Legionella* spp.	Spain*	Environmental screen	Broth dilution	Resistance to TTPC. DDAC, DBNPA, H_2O_2 + $AgNO_3$, THPS, NaCOl, BZK and MCI	[77]
Hospital	Sink	*Klebsiella pneumoniae*	France	Outbreak investigation	MALDI	2 OXA-48 positive isolates	[97]
Hospital	Sink drain<br>Tap water	*S. maltophila*<br>*Chryseobacterium* spp.<br>*Sphingomonas paucimobilis*<br>*E. cloacae*<br>*Methylobacterium* spp.<br>*Bosea* spp.<br>*Acinetobacter johnsonii*<br>*Acidovorax* spp.<br>*P. aeruginosa*<br>*Mycobacterium chelonae*	Canada*	Environmental screen	PCR	*tetG* found in 1 water sample<br>*ermF* found in 1 biofilm sample<br>*ermX* found in 1 biofilm and 1 water sample	[98]
Hospital	Sinks<br>Faucets<br>Drains	*Elizabethkingia meningoseptica*	China	Environmental screen	Broth microdilution, PCR	Resistance reported as whole environmental data	[99]
Hospital	Hands free taps	*A. baumannii*	Japan*	Outbreak investigation	PCR, MLST	AMK and CIP resistant strain	[103]
Hospital	Sink<br>Tap	*Pseudomonas aeruginosa*	India*	Clinical isolates v environmental isolates	Disc diffusion	Environmental resistance profiles reported as total with clinical isolates	[106]
Hospital	Sink	*E. cloacae*	England*	Outbreak investigation	Rosco tablets	Resistance profile for environmental isolates not reported	[107]
Hospital	Sink	*P. aeruginosa*	India*	Environmental screen	Disc diffusion	Resistance profiles reported as whole environmental data	[108]
Hospital	Drain-pipe leak	*E. cloacae*	India	Outbreak investigation	Disc diffusion	Resistant to: AMP, TSU, GEN, CTX, OFX	[109]
Hospital	Water<br>Drain<br>Basin<br>Shower<br>Toilet	*E. cloacae*	France	Outbreak investigation	Disc diffusion	Resistance seen to ADBAC and DDAC from shower and sink drains	[110]
Hospital	Sink	*K. pneumoniae*<br>*E.cloacae* complex<br>*Citrobacter freundii*<br>*Klebsiella oxytoca*<br>*Escherichia coli*	Belgium	Outbreak investigation	Disc diffusion, Xpert carba-R assay	NDM, KPC and OXA-48 resistance found	[111]
Dental office	Water	*P. aeruginosa*	Brazil	Environmental screen	Disc diffusion	Resistance reported as whole environmental data	[112]
Hospital	Water	*Pseudomonas* spp.	USA	Equipment screen	Disc diffusion	Resistant to 5-10% ethylene glycol	[113]
Hospital	Water	*E. coli*	Ghana*	Environmental screen	Disc diffusion	Resistant to:<br>AMP<br>TET	[114]

						CHL<br>TSU	
Hospital	Sink	*E. coli*<br>*K. pneumoniae*<br>*Klebsiella ozenae*<br>*Proteus mirabilis*<br>*E. cloacae*<br>*Enterobacter aerogenes*<br>*Providencia stuartii*<br>*Citrobacter spp.*	Ethiopia	Environmental screen	Disc diffusion	All ESBL resistant to: CPR, CPD, CAZ, CRO and AMC	[115]
Hospital	Sink<br>Nebulizer<br>Water	*P. aeruginosa*<br>*Acinetobacter anitratus*<br>*E. coli*<br>*Proteus* spp.<br>*Enterobacter* spp.	UK	Environmental screen	Disc diffusion	Resistance reported as whole environmental data	[116]
Hospital	Sink	*Klebsiella* spp.<br>*E. coli*<br>*Citrobacter* spp.<br>*Enterobacter* spp.<br>*Proteus* spp.<br>*Aeromonas hydrophilia*	UK	Outbreak investigation	Disc diffusion	Resistance reported as whole environmental data	[117]
Hospital	Sink<br>Water	*K. pneumoniae*	Nigeria	Outbreak investigation	Disc diffusion	Resistance profile: AMC, CPR, CAZ, CRO, GEN and CIP	[118]
Hospital	Shower head<br>Shower outlets<br>Basin<br>Toilet	*S. maltophila*	Germany	Outbreak investigation	Disc diffusion	Resistance to TMP-SMX	[119]
Hospital	Tap	157 species	Saudi Arabia*	Environmental screen	Microscan	Resistance reported as whole environmental data	[120]
Hospital	Drain<br>Sink<br>Shower	*K. oxytoca*	Austria*	Outbreak investigation	Microarray	blaKPC and blaTEM found in all isolates. Sink isolates resistant to carbapenems	[121]
Hospital	Water	*Burkholderia* spp.	Spain	Clinical isolates v environmental isolates	E-test	Resistance reported as whole environmental data	[122]
Hospital	Sink faucets<br>Sink drains	*Acinetobacter baumannii*	Singapore	Outbreak investigation	Disc diffusion, Whole genome sequencing	No water source isolate identified as resistant	[123]
Hospital	Sink	*P. aeruginosa*<br>*Serratia liquefaciens*<br>*Acinetobacter* spp.<br>*Serratia marcescens*<br>*E. meningoseptica*	Oman	Environmental screen	DST	Resistance reported as whole environmental data	[124]

		Chryseobacterium indologenes					
Hospital	Drain<br>Water	*S. maltophila*	Thailand	Clinical isolates v environmental isolates	Disc diffusion	Greatest resistance seen to CHL, AMK and CST	[125]
Hospital	Sink	*Mycobacterium* spp.	Portugal*	Environmental search	Disc diffusion	Resistance to CIP, CLR, IMI, TOB	[126]
Hospital	Water	Enterococci	India	Environmental water screen	Disc diffusion	Resistance reported as whole environmental data	[127]
Hospital	Sink	*S. aureus*<br>*K. pneumoniae*<br>*Acinetobacter* spp.<br>*P. aeruginosa*<br>*Enterobacter* spp.	Malaysia	Environmental screen	Disc diffusion	Resistance reported as whole environmental data	[128]
Hospital	Water	*Aeromonas* spp.	Nepal	Clinical isolates v environmental isolates	Disc diffusion	25% of *Aeromonas* spp. Isolates resistant to NAL	[129]
Hospital	Water<br>Sinks<br>Showers	*S. marcescens*	Canada*	Outbreak investigation	Agar dilution	Sink isolates resistant to AMP, CEF, FOX, TIC, TSU, NIT, KAN, GEN and TOB.<br>Produced ANT2' and AAC6'	[130]
Hospital	Water	*E. cloacae*	China	Outbreak investigation	Disc diffusion	Resistance reported as whole environmental data	[131]
Hospital	Sinks<br>Nasogastric water	*A. baumannii*	Taiwan	Outbreak investigation	Disc diffusion, Agar dilution	Outbreak organism found on sink and in nasogastric water	[132]
Hospital	Sink<br>Water<br>Hydrotherapy tanks	*P. aeruginosa*	India*	Clinical isolates v environmental isolates	Disc diffusion	Two MBL *P. aeruginosa* isolates resistant to IMI	[133]
Hospital	Faucet<br>Basin	*K. pneumoniae*	Algeria	Environmental screen	Disc diffusion, PCR	All isolates resistant to AMX, CTX, CRO and TIM	[134]
Hospital	Water<br>Sink trap<br>Tap surface	*P. aeruginosa*	China*	Environmental screen	Disc diffusion	No resistant isolates from water sources	[135]

In country, where the study location was not specified in the article, '*' was denoted with the country of the authors.

∞ Abbreviations: DST, direct susceptibility testing; MALDI-TOF, matrix-assisted laser desorption/ionization-time of flight; MLST, multilocus sequencing typing; PCR, polymerase chain reaction; PFGE, pulse field gel electrophoresis

∧ Abbreviations: CRE, carbapenem-resistant *Enterobacteriaceae;* Zn, zinc; Cu, copper; Cd, cadmium; Hg, mercury; MRSA, methicillin resistant *Staphylococcus aureus*; MDR, multi drug resistant; ADBAC, benzalkonium chloride; AMC, amoxicillin; AMK, amikacin; AMP, ampicillin; ATM, aztreonam; BZK, Benzalkonium chloride; CAZ, ceftazidime; CEF, cephalothin; CHL, chloramphenicol; CIN, cinoxacin; CIP, ciprofloxacin; CLR, clarithromycin; CPD, cefpodoxime; CPR, cefprozil; CRO, ceftriaxone; CST, colistin; CTX, cefotaxime; DBNPA, 2,2-dibromo-3-nitrilopropionamide; DDAC, didecyldimethyl ammonium chloride; FEP, cefepime ; FOX, cefoxitin; GEN, gentamicin; H2O2 + AGNO3, Hydrogen peroxide + Silver nitrate, H_2O_2 + $AgNO_3$; IMI, imipenem ; KAN, kanamycin;

MCL, methylene chloride; MEM, meropenem; NAOCL, Sodium hypochlorite; NET, netilmicin; NIT, nitrofurantoin; OFX, ofloxacin; PIP, piperacillin; SAM, ampicillin-sulbactam; STR, streptomycin; TET, tetracycline; THPS, tetrakis (hydroxymethyl)phosphonium sulfate; TIC, ticarcillin; TMP-SMX, trimethoprim + sulfamethoxazole; TOB, tobramycin; TSU, cotrimoxazole; TTPC, tributyl tetradecyl phosphonium chloride; TZP, piperacillin-tazobactam

References

106. Aditi; Shariff, M.; Chhabra, S.K.; Rahman, M.U. Similar virulence properties of infection and colonization associated *Pseudomonas aeruginosa*. *J. Med. Microbiol.* **2017**, *66*, 1489–1498, doi:10.1099/jmm.0.000569.
107. Andersen, B.M.; Sørlie, D.; Hotvedt, R.; Almdahl, S.M.; Olafsen, K.; George, R.; Gilfillian, A. Multiply beta-lactam resistant *Enterobacter cloacae* infections linked to the environmental flora in a unit for cardiothoracic and vascular surgery. *Scand. J. Infect. Dis.* **1989**, *21*, 181–191, doi:10.3109/00365548909039967.
108. Babu, K.V.Y.; Kumara, A.; Vijayanath, V. Study of imipenem resistant metallo-beta-lactamase positive *Pseudomonas aeruginosa* from different hospital environmental sources. *J. Pure Appl. Microbiol.* **2011**, *5*, 195–203.
109. Banerjee, G.; Ayyagari, A.; Prasad, K.N.; Dhole, T.N.; Singh, S.K. Nosocomial infection due to *Enterobacter cloacae* in a tertiary care hospital in northern India. *Indian J. Med. Res.* **1996**, *103*, 58–61.
110. Chapuis, A.; Amoureux, L.; Bador, J.; Gavalas, A.; Siebor, E.; Chretien, M.L.; Caillot, D.; Janin, M.; de Curraize, C.; Neuwirth, C. Outbreak of extended-spectrum beta-lactamase producing *Enterobacter cloacae* with high MICs of quaternary ammonium compounds in a hematology ward associated with contaminated sinks. *Front. Microbiol.* **2016**, *7*, doi:10.3389/fmicb.2016.01070.
111. De Geyter, D.; Blommaert, L.; Verbraeken, N.; Sevenois, M.; Huyghens, L.; Martini, H.; Covens, L.; Piérard, D.; Wybo, I. The sink as a potential source of transmission of carbapenemase-producing *Enterobacteriaceae* in the intensive care unit. *Antimicrob. Resist. Infect. Control* **2017**, *6*, doi:10.1186/s13756-017-0182-3.
112. De Oliveira, D.V.; Nunes, L.S.; Barth, A.L.; Van Der Sand, S.T. Genetic background of β-lactamases in *Enterobacteriaceae* isolates from environmental samples. *Microb. Ecol.* **2017**, *74*, 599–607, doi:10.1007/s00248-017-0970-6.
113. Du Moulin, G.C.; Doyle, G.O.; MacKay, J.; Hedley-Whyte, J. Bacterial fouling of a hospital closed-loop cooling system by *Pseudomonas* sp. *J. Clin. Microbiol.* **1981**, *13*, 1060–1065.
114. Duedu, K.O.; Offei, G.; Codjoe, F.S.; Donkor, E.S. Multidrug resistant enteric bacterial pathogens in a psychiatric hospital in ghana: Implications for control of nosocomial infections. *Int. J. Microbiol.* **2017**, *2017*, doi:10.1155/2017/9509087.
115. Engda, T.; Moges, F.; Gelaw, A.; Eshete, S.; Mekonnen, F. Prevalence and antimicrobial susceptibility patterns of extended spectrum beta-lactamase producing *Enterobacteriaceae* in the University of Gondar Referral Hospital environments, northwest Ethiopia. *BMC Res. Notes* **2018**, *11*, doi:10.1186/s13104-018-3443-1.
116. Fujita, K.; Lilly, H.A.; Ayliffe, G.A.J. Spread of resistant Gram-negative bacilli in a burns unit. *J. Hosp. Infect.* **1982**, *3*, 29–37, doi:10.1016/0195-6701(82)90028-7.
117. Hart, C.A. Nosocomial gentamicin- and multiply-resistant *Enterobacteria* at one hospital. 1. Description of an outbreak. *J. Hosp. Infect.* **1982**, *3*, 15–28, doi:10.1016/0195-6701(82)90027-5.
118. Iregbu, K.C.; Anwaal, U. Extended spectrum beta-lactamase-producing *Klebsiella pneumoniae* septicaemia outbreak in the neonatal intensive care unit of a tertiary hospital in Nigeria. *Afr. J. Med. Med. Sci.* **2007**, *36*, 225–228.
119. Kampmeier, S.; Pillukat, M.H.; Pettke, A.; Kossow, A.; Idelevich, E.A.; Mellmann, A. Evaluation of a *Stenotrophomonas maltophilia* bacteremia cluster in hematopoietic stem cell transplantation recipients using whole genome sequencing. *Antimicrob. Resist. Infect. Control* **2017**, *6*, doi:10.1186/s13756-017-0276-y.
120. Khan, M.A.; Al-Motair, K.A.; Alenezi, M.M.; Altheban, A.S.; Hammam, S.A.; Al-Mogbel, M.S. Nosocomial pathogens—A single center study in Saudi Arabia. *J. Pure Appl. Microbiol.* **2018**, *12*, 1411–1416, doi:10.22207/JPAM.12.3.44.
121. Leitner, E.; Zarfel, G.; Luxner, J.; Herzog, K.; Pekard-Amenitsch, S.; Hoenigl, M.; Valentin, T.; Feierl, G.; Grisold, A.J.; Högenauer, C.; et al. Contaminated handwashing sinks as the source of a clonal outbreak of KPC-2-producing *Klebsiella oxytoca* on a hematology ward. *Antimicrob. Agents Chemoth.* **2015**, *59*, 714–716, doi:10.1128/AAC.04306-14.
122. Medina-Pascual, M.J.; Valdezate, S.; Villalon, P.; Garrido, N.; Rubio, V.; Saez-Nieto, J.A. Identification, molecular characterisation and antimicrobial susceptibility of genomovars of the *Burkholderia cepacia* complex in Spain. *Eur. J. Clin. Microbiol. Infect. Dis.* **2012**, *31*, 3385–3396, doi:10.1007/s10096-012-1707-6.
123. Ng, C.; Tay, M.; Tan, B.; Le, T.H.; Haller, L.; Chen, H.J.; Koh, T.H.; Barkham, T.M.S.; Gin, K.Y.H. Characterization of metagenomes in urban aquatic compartments reveals high prevalence of clinically relevant antibiotic resistance genes in wastewaters. *Front. Microbiol.* **2017**, *8*, doi:10.3389/fmicb.2017.02200.

124. Nzeako, B.C.; Al Daughari, H.; Al Lamki, Z.; Al Rawas, O. Nature of bacteria found on some wards in Sultan Qaboos University Hospital, Oman. *Br. J. Biomed. Sci.* **2006**, *63*, 55–58, doi:10.1080/09674845.2006.11732720.
125. Paopradit, P.; Srinitiwarawong, K.; Ingviya, N.; Singkhamanan, K.; Vuddhakul, V. Distribution and characterization of *Stenotrophomonas maltophilia* isolates from environmental and clinical samples in Thailand. *J. Hosp. Infect.* **2017**, *97*, 185–191, doi:10.1016/j.jhin.2017.06.006.
126. Pereira, S.G.; Cardoso, O. Mobile genetic elements of *Pseudomonas aeruginosa* isolates from hydrotherapy facility and respiratory infections. *Clin. Microbiol. Infect.* **2014**, *20*, O203–O206, doi:10.1111/1469-0691.12359.
127. Peter, A.; Mathew, J.; Zacharia, S. Antibiotic resistant enterococci from drinking water sources. *Asian J. Pharmaceut. Clin. Res.* **2012**, *5*, 158–160.
128. Phoon, H.Y.P.; Hussin, H.; Hussain, B.M.; Lim, S.Y.; Woon, J.J.; Er, Y.X.; Thong, K.L. Distribution, genetic diversity and antimicrobial resistance of clinically important bacteria from the environment of a tertiary hospital in Malaysia. *J. Glob. Antimicrob. Resist.* **2018**, *14*, 132–140, doi:10.1016/j.jgar.2018.02.022.
129. Pokhrel, B.M.; Thapa, N. Prevalence of *Aeromonas* in different clinical and water samples with special reference to gastroenteritis. *Nepal Med. Coll. J.* **2004**, *6*, 139–143.
130. Simor, A.E.; Wilcox, L. Molecular and epidemiologic study of multiresistant *Serratia marcescens* infections in a spinal cord injury rehabilitation unit. *Infection Control Hosp. Epidemiol.* **1988**, *9*, 20–27, doi:10.1086/645728.
131. Wang, C.C.; Chu, M.L.; Ho, L.J.; Hwang, R.C. Analysis of plasmid pattern in paediatric intensive care unit outbreaks of nosocomial infection due to *Enterobacter cloacae*. *J. Hosp. Infect.* **1991**, *19*, 33–40, doi:10.1016/0195-6701(91)90126-S.
132. Wang, S.H.; Sheng, W.H.; Chang, Y.Y.; Wang, L.H.; Lin, H.C.; Chen, M.L.; Pan, H.J.; Ko, W.J.; Chang, S.C.; Lin, F.Y. Healthcare-associated outbreak due to pan-drug resistant *Acinetobacter baumannii* in a surgical intensive care unit. *J. Hosp. Infect.* **2003**, *53*, 97–102, doi:10.1053/jhin.2002.1348.
133. Yogeesha Babu, K.V.; Vijayanath, V.; Niranjan, H.P.; Anitha, M.R. Study of imipenem resistant metallo-beta-lactamase positive *Pseudomonas aeruginosa* from burns wound infections, environmental sources and impact of infection control measures in a burns care center. *J. Pure Appl. Microbiol.* **2011**, *5*, 695–703.
134. Zenati, K.; Sahli, F.; Garcia, V.; Bakour, S.; Belhadi, D.; Rolain, J.M.; Touati, A. Occurrence and clonal diversity of multidrug-resistant *Klebsiella pneumoniae* recovered from inanimate surfaces in Algerian hospital environment: First report of *armA*, *qnrB* and *aac(6′)-Ib-cr* genes. *J. Glob. Antimicrob. Resist.* **2017**, *10*, 148–153, doi:10.1016/j.jgar.2017.05.015
135. Zhou, Z.; Hu, B.; Gao, X.; Bao, R.; Chen, M.; Li, H. Sources of sporadic Pseudomonas aeruginosa colonizations/infections in surgical ICUs: Association with contaminated sink trap. J. Infect. Chemoth. 2016, 22, 450–455, doi:10.1016/j.jiac.2016.03.016
